# Skip Island Craniotomy: A Technique for Managing Superior Sagittal Sinus Injury in Emergency Neurosurgery

**DOI:** 10.7759/cureus.82050

**Published:** 2025-04-10

**Authors:** Azam A Baig, Luke Galloway, Wai C Soon, Paul Dias, Hari Krovvidi, Thomas Land, Ramesh Chelvarajah

**Affiliations:** 1 Department of Neurosurgery, Queen Elizabeth Hospital Birmingham, Birmingham, GBR; 2 Department of Anesthesiology, Queen Elizabeth Hospital Birmingham, Birmingham, GBR

**Keywords:** brain bleed, penetrating traumatic head injury, superior sagittal sinus, trauma, traumatic brain injury

## Abstract

Superior sagittal sinus (SSS) injury can be a life-threatening condition. It is rarely injured by means of penetrating and nonpenetrating traumatic brain injury (TBI). Injury to the SSS can be a surgical challenge and thus provides a conundrum to neurosurgeons on its management in an acute emergency setting.

We present a series of two cases that were successfully treated by a novel skip island craniotomy technique after suffering a penetrating and nonpenetrating TBI-related SSS injury, respectively.

Both patients had a short period of ITU stay before being managed on the neurosurgery ward and went on to have no neurological deficits. The operative wounds healed well, and overall cosmesis was unaffected. Postoperative computed tomography head scans with 3D reconstruction in bone window demonstrate the island-like pattern with interval burr holes.

## Introduction

Superior sagittal sinus (SSS) injury can be a life-threatening condition. It is not commonly encountered in penetrating or nonpenetrating traumatic brain injury (TBI) [1]. On the rare occasion that it occurs, after direct penetrating injury or fracture-related injury, it poses a conundrum to neurosurgeons in its management.

We present a series of two cases that were successfully treated by a skip island craniotomy technique after suffering a penetrating and nonpenetrating TBI-related SSS injury, respectively. To our knowledge, this technique has not previously been described in the literature.

## Case presentation

Case 1

**Figure 1:**
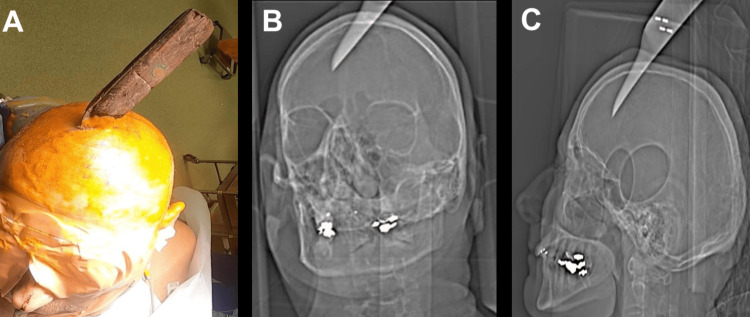
Preoperative photograph of case 1 in the operating room (A). Topograph skull X-rays of case 1 depicting bladed article penetrating the skull (B) and crossing the midline (C)

**Figure 2:**
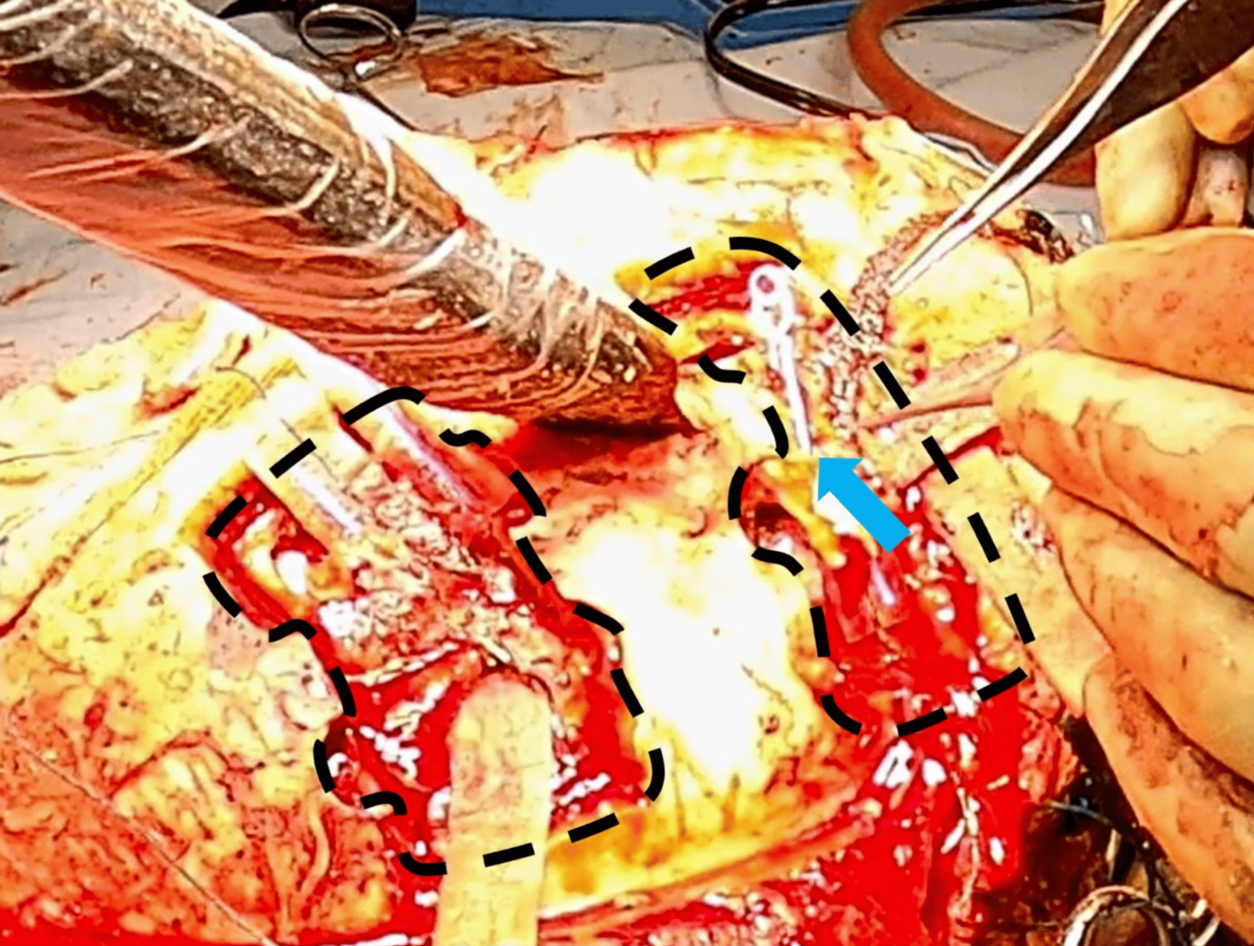
Intraoperative photograph demonstrating completed anterior and posterior craniotomies (black dotted lines), bladed article in situ, and large aneurysm clip (blue arrow) clamping the anterior segment of the superior sagittal sinus

**Figure 3:**
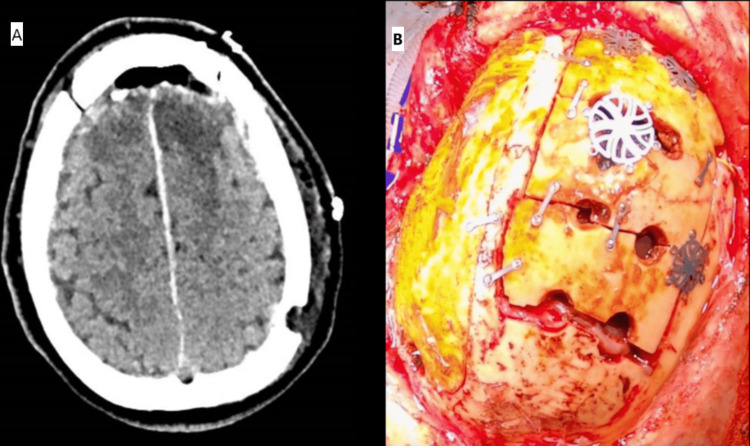
Postoperative plain CT head (A) of case 1 showing satisfactory appearance and intraoperative photograph (B), revealing midline island-like craniotomies over the superior sagittal sinus CT: computed tomography

A 61-year-old male patient, with a history of psychosis, presented with a self-inflicted penetrating injury to the top of his head. A kitchen knife was in situ, entering at the level of the coronal suture approximately in the midline (Figure 1A). On initial assessment, he was hemodynamically stable with a Glasgow Coma Scale (GCS) of 15 and no neurological deficit. Topograph skull X-rays revealed the penetrating bladed article (Figures 1B, 1C). The computed tomography (CT) head showed the bladed article in situ entering the skull at the level of the coronal suture just to the left of the midline and protruding 57 mm anteromedially across the interhemispheric fissure with the tip entering the right frontal lobe. In addition, there were left convexity and interhemispheric subdural hematomas with a midline shift of 7 mm. He was commenced on an antiepileptic agent, antibiotics, and a CT angiogram, and a venogram was performed. These investigations showed obvious penetrating laceration of the SSS but no evidence of active bleeding. He was taken to the theater directly for left-sided and midline craniotomies, evacuation of acute subdural hematoma, and removal of midline foreign body. Left-sided craniotomy was performed first, with the evacuation of the convexity subdural hematoma. A midline craniotomy was performed as a strip with three sections (anterior, middle, and posterior). The anterior and posterior sections of the midline craniotomy were removed with a craniotome, leaving the middle section intact laterally to the adjacent skull on both sides, thereby supporting the knife. Large aneurysm clips were placed across the SSS anterior and posterior to the knife blade (Figure 2), then the middle bone flap was undertaken while the knife was carefully supported. The knife and middle section craniotomy were removed together with the finding of complete oblique transection of the SSS. The tip of the knife had caused a small laceration of the left superior frontal gyrus and entered the interhemispheric fissure; however, the pericallosal and callosomarginal vessels were intact. There was excessive tension on the transected ends of the sinus for an attempt to anastomose, and as the sinus transection was at the level of the coronal suture, we decided to ligate the two ends with silk sutures. A right frontal intraparenchymal intracranial pressure monitor and subgaleal gravity drain were inserted. Burr hole covers and small dog-bone-shaped titanium plates were used to reconstruct the bone flaps, and postoperative plain CT head showed satisfactory appearance (Figure 3). Our patient was kept intubated for 24 hours and started on prophylactic low-molecular-weight heparin the next day.

Case 2

**Figure 4:**
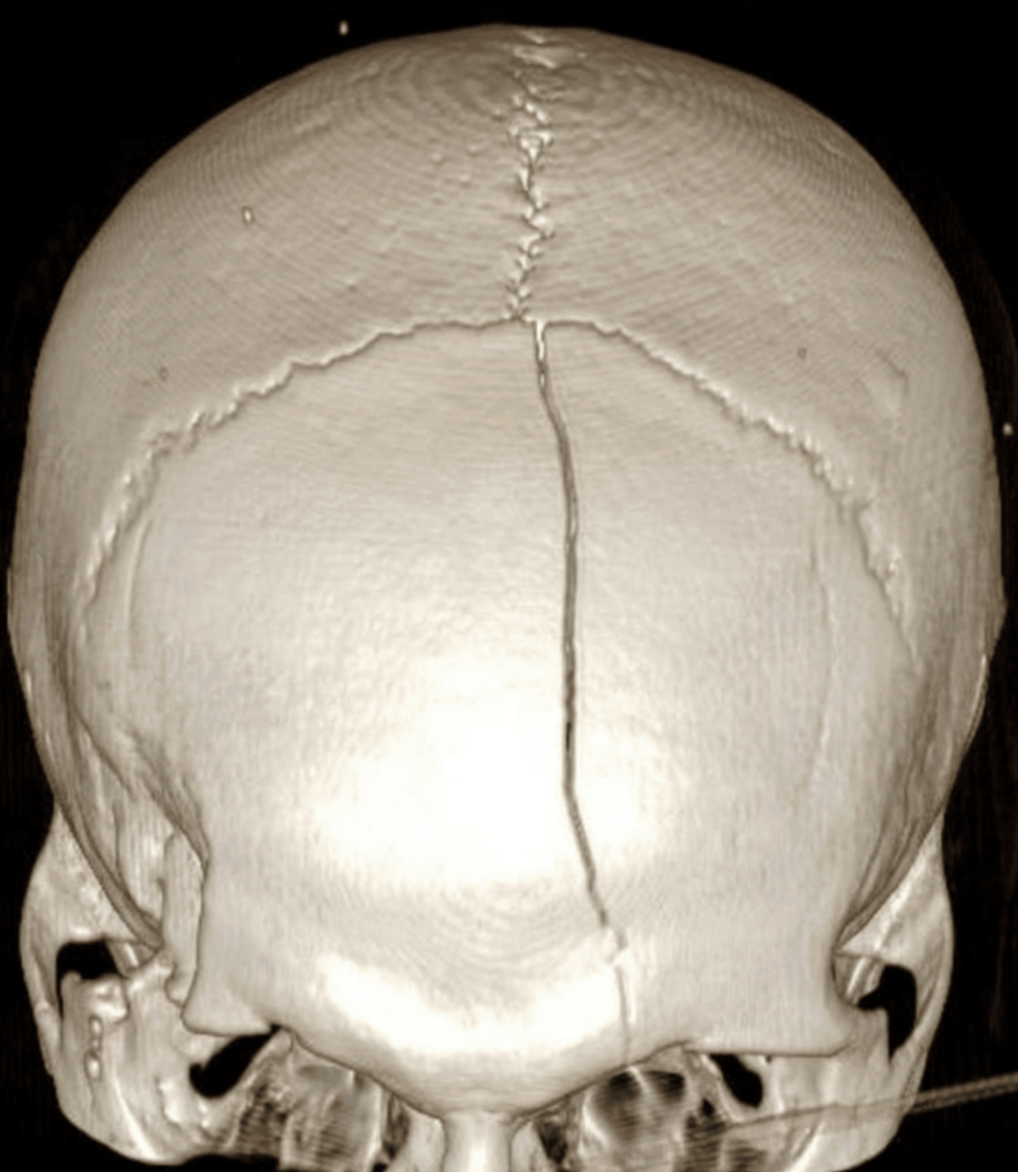
3D reconstruction of case 2 trauma CT head showing large linear frontal bone fracture 3D: three dimensional; CT: computed tomography

**Figure 5:**
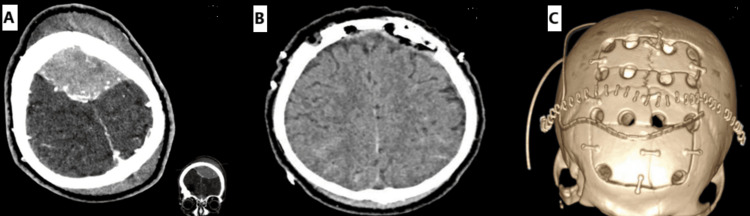
CT head axial cut of case 2 showing large midline bifrontoparietal EDH and fracture-related SSS injury and tear with corresponding coronal view (A). Plain postoperative CT head of case 2 revealing evacuation of EDH and repair of the SSS (B). 3D reconstruction of (B) illustrating island-like craniotomies over the SSS (C) CT: computed tomography; EDH: extradural hematoma; SSS: superior sagittal sinus

A 35-year-old male patient presented after a fall following a spontaneous generalized tonic-clonic seizure. GCS at scene was 13 and dropped to 10 on arrival at the hospital. Plain CT head showed a large midline bifrontoparietal extradural hematoma (EDH) causing significant mass effect onto the left frontal region with modest bifrontobasal and left frontopolar contusions and traumatic subarachnoid hemorrhage. Bone window revealed a left frontal linear fracture (Figure 4) extending from coronal-sagittal suture confluence to left frontal air sinus, both walls with no displacement, and a right lateral orbital bar/supraorbital lateral frontal fracture with marginal displacement and underlying small fracture hematoma, which was managed conservatively initially. Four-hour interval repeat CT head showed a significant increase in EDH, and he was subsequently taken to the theater (Figure 5A). The skip island craniotomy required the surgeon to perform three midline craniotomies, with the aim of gaining control of the SSS both anteriorly and posteriorly. A middle window of intact bone was left overlying the suspected location of SSS injury. The anterior and posterior craniotomies or islands were completed. Bilateral dural opening was performed to visualize the falx and SSS anteriorly and posteriorly. Vascular cross-clamps were then placed on the SSS anteriorly and posteriorly. With the cross-clamps applied, the middle island of bone was removed, and with control of the SSS, primary repair was performed in the controlled conditions. Postoperative CT head and early serial CT venograms showed good evacuation of EDH (Figure 5B). There is no suggestion of venous sinus thrombosis with expected focal narrowing of SSS at the point of repair of fracture-related sinus injury and tears. Postoperative CT head scan (Figure 5C) was then reconstructed in bone window to illustrate an island-like pattern with interval burr holes.

Both patients had a short temporary period of ITU stay before being managed on the neurosurgery ward and did not suffer neurological deficits. Low-molecular-weight heparin was initiated postoperatively for prophylaxis. The operative wounds healed well and overall cosmesis was unaffected.

## Discussion

Blunt TBIs can cause traumatic injury of major cerebral venous sinuses by skull fractures extending to a dural sinus or jugular bulb [2]. Meanwhile, in penetrating TBI, the foreign body can cause direct injury to a venous sinus [3]. Although knife wounds to the head are low energy/velocity, they may penetrate the skull and injure the cortex or vascular structures, resulting in intracranial hemorrhage and possible death. Due to the effective barrier provided by the adult calvarium, most injuries occur through the orbital or temporal regions where bony layers are thin, and thus, stab injury to the midline is uncommon [4].

Management of venous sinus injury ideally involves exposure of the sinus both proximally and distally to the site of injury. The bone flap should extend well beyond the edge of any fracture, which will require craniotomy across both sides of the sinus, and care must be taken to avoid further injury [5]. Nevertheless, studies have documented significant patient morbidity following traditional operative methods, including hemorrhages, infection, wound complications, air embolism, and severe disability [6,7]. We, however, have demonstrated that appropriate exposure through a staged craniotomy to gain vascular control can be achieved for injury and repair. This process is essential for the removal of penetrating objects associated with venous sinus injuries and their lacerations, ultimately leading to satisfactory outcomes. CT venogram is recommended in the initial trauma workup and postsurgery to evaluate the venous sinus injury and repair [8].

## Conclusions

TBI-related SSS injury, although rare, can pose difficult management challenges to neurosurgeons. Appropriate exposure with a staged craniotomy to gain vascular control are essential in these cases with associated venous sinus injury. We propose this novel technique as safe, straight forward to perform, and one that can lead to satisfactory outcomes.
